# Scheduling multi-task jobs with extra utility in data centers

**DOI:** 10.1186/s13638-017-0986-0

**Published:** 2017-11-25

**Authors:** Xiaolin Fang, Junzhou Luo, Hong Gao, Weiwei Wu, Yingshu Li

**Affiliations:** 1 School of Computer Science and Engineering, Southeast University, Nanjing, China; 2 School of Computer Science and Technology, Harbin Institute of Technology, Harbin, China; 3 Department of Computer Science, Georgia State University, Atlanta, USA

**Keywords:** Multi-task jobs, Extra utility, Scheduling

## Abstract

This paper investigates the problem of maximizing utility for job scheduling where each job consists of multiple tasks, each task has utility and each job also has extra utility if all tasks of that job are completed. We provide a 2-approximation algorithm for the single-machine case and a 2-approximation algorithm for the multi-machine problem. Both algorithms include two steps. The first step employs the Earliest Deadline First method to compute utility with only extra job utility, and it is proved that it obtains the optimal result for this sub-problem. The second step employs a Dynamic Programming method to compute utility without extra job utility, and it also derives the optimal result. An approximation result can then be obtained by combining the results of the two steps.

## Introduction

Job scheduling is a widely studied topic in computer science. Many systems such as parallel and distributed computing, cloud computing, workforce management, energy management, and network communications require scheduling of jobs [1–5]. There are many studies designing efficient approaches to solve the job scheduling problem so as to improve the resultant performance subject to the resource constraints [6–9].

Many applications prefer to divide large jobs into multiple small tasks to better utilize the limited resources and provide better service quality. As stated in [10], most interactive services such as web search, social networks, online gaming, and financial services now are heavily dependent on computations at data centers because their demands for computing resources are both huge and dynamic. Interactive services are time-sensitive as users expect to receive a complete or possibly partial response within a short period of time. Thus, a job should be preemptive and it can be divided into many small tasks (we call this as multi-task problem) in order to provide interactive services and improve the utilization ratio of the computing resources.

We study the multi-task job scheduling problem in this paper. Usually, the aim of multi-task job scheduling is to maximize the profit or minimize the cost while subject to the resource and deadline constraints. This paper also studies the profit maximization problem for multi-task job scheduling where each job has a starting time and an ending time. The profit is called *utility* in this paper. The utility of a task or job can be obtained only if the task or job is completed. Most state of art works study the problem considering either the utility of the tasks or the utility of the jobs. Few works consider both the utility of the tasks and jobs. In this paper, we study the problem of multi-task job scheduling at a data center with the goal of maximizing the total utility of all the jobs, where each job is decomposed into multiple tasks, and both a job and a task have their own utility. That is, each task has its own utility and each job also has an extra utility which can only be obtained when all its tasks are completed.

The problem investigated in this paper is particularly challenging because it is quite difficult to decide whether it is better to schedule a job as a whole or to schedule the tasks of the job separately. Furthermore, it is difficult to make correct decisions for current jobs because the requirements of the incoming jobs are unknown.

We first study the single-machine problem where there is only one machine that can be used. The single-machine problem expects a method to schedule the jobs on one machine while satisfying the resource and deadline constraints. We then study the multi-machine problem where multiple machines can be employed. Because of the NP-completeness of the problem, we present two corresponding 2-approximation algorithms for the two problems.

For simplicity, we only consider the problem where every task has uniform resource requirement, but the utility of the tasks could be different. Tasks have arbitrary resource requirements will be studied in the future work. Our contributions are summarized as follows.

- To the best of our knowledge, this is the first work to study the problem considering both task utility and job utility.
- A 2-approximation algorithm is provided for the single-machine problem. This algorithm includes an Earliest Deadline First (EDF) scheduling and a Dynamic Programming (DP) algorithm.
- Another 2-approximation algorithm is provided for the multi-machine problem. Similar to the algorithm for the single-machine problem, this algorithm also employs an EDF scheduling and a DP algorithm.

The rest of the paper is organized as follows. Section 2 introduces the related works. Section 3 presents the problem formulation. Section 4 studies the single-machine problem. The multi-machine problem is studied in Section 5. And Section 6 concludes the paper.

## Related works

The job scheduling problem can be classified into multiple classes, such as single or multiple tasks, single or multiple machines, and identical or unrelated machines. Usually, the input of the problem involves *n* jobs and *k* machines. Each job is associated with a release time, a deadline, a weight, and a processing time on each machine. The goal is to find a non-preemptive schedule that maximizes the weight of the jobs subject to their respective deadlines. Garey and Johnson [11, 12] show that the simplest instance of the decision problem corresponding to this problem is NP-complete.

Bar-Noy et al. [13, 14] study the scheduling problem where each job includes a single task. The authors present a 3-approximation algorithm using the local ratio technique. For arbitrary job weights and a single machine, an LP formulation achieves a 2-approximation for polynomially bounded integral input and a 3-approximation for arbitrary input. For unrelated machines, the factors are 3 and 4, respectively. Because of the high time complexity of the LP-based method, Bar-Noy et al. [13] also provide a combinatorial approximation algorithm whose approximation factor is $3+2\sqrt {2}$. Independently, Calinescu et al. [15] designed a 3-approximation algorithm via rounding linear programming solutions.

The preemptive version of the single-task problem for a single machine was studied by Lawler [16]. For identical job weights, Lawler showed how to apply the dynamic programming techniques to solve the problem in polynomial time. The same techniques are employed to obtain a pseudopolynomial algorithm for the NP-hard variant in which the weights are arbitrary. Lawler [17] also designed polynomial time algorithms that solve the problem in two special cases: (i) the time windows in which jobs can be scheduled are nested, and (ii) the weights and processing times are in opposite order. Kise et al. [18] showed how to solve the special case where the release times and deadlines are similarly ordered.

Some works [19–22] study the problem where each job has multiple tasks, which is called the SplitJob problem. In the SplitJob problem, a task does not have a window within which the tasks can be scheduled. That is, the tasks can only be decided to be scheduled or not. The unit height case of the basic SplitJob problem has been addressed by finding the maximum weight independent sets in interval graphs [19, 20]. Bar-Yehuda et al. [21] present a (2*r*)-approximation algorithm, where *r* is the number of the tasks in a job. They also proved a hardness result indicating it is NP-hard to approximate the problem within a factor of *O*(*r*/log*r*). Thus, their approximation ratio is near-optimal. Bar-Yehuda and Rawitz [22] studied the uniform case of the basic SplitJob problem and derived a (6*r*)-approximation algorithm by utilizing the fractional local ratio technique.

Venkatesan et al. [23] study the problem of maximizing the throughput of jobs where each job consists of multiple tasks. Different from the SplitJob problem, each task has a window where the task can be scheduled any time within the window subject to the processing length. The algorithm presented in [23] is an LP-based algorithm which gives 8*r*-approximation.

All the above works either consider the utility of tasks or the utility of jobs. In this paper, we study the problem where each job consists of multiple tasks, each task has utility, and each job has extra utility if all its tasks are completed.

A closely related problem is considered by Zheng et al. in [10] which study the problem of scheduling interactive jobs at a data center with the goal of maximizing the total utility of all the jobs. In their problem, the utility of a job is a function of the completed workload of that job. That is, the utility of a job varies when the completed workload of that job increases. The presented function in their work is nonlinear and concave. If the scheduling can be preemptive, then the authors can provide an optimal solution to solve the problem.

## System model and problem formulation

### System model

Assume there are *m* physical machines {*M*_1_,*M*_2_,…,*M*_*m*_} and *n* jobs {*J*_1_,*J*_2_,…,*J*_*n*_} in the data centers. Each job *J*_*i*_ has a starting time *s*_*i*_ and an ending time *e*_*i*_, i.e., *J*_*i*_=[*s*_*i*_,*e*_*i*_], which is called the *processing interval*. Each job needs to be completed within its own processing interval. Each job *J*_*i*_ consists of multiple tasks $\{T_{i1},T_{i2},\dots,T_{in_{i}}\}$, where *n*_*i*_ is the number of the tasks involved in job *J*_*i*_. Each task *T*_*ij*_ has a *processing time* of length 1 and a utility *u*_*ij*_, which means it needs 1 machine to take 1 unit of time to complete task *T*_*ij*_, and if the task is completed, then the utility is *u*_*ij*_. With the above assumption, it can be easily found that each task *T*_*ij*_ must be completed within the processing interval [*s*_*i*_,*e*_*i*_]; otherwise, the task is dropped. We define the *assignment* of a task *T*_*ij*_ as *∅* or a sub-interval *I*_*ij*_ of 1 unit of length within the processing interval [*s*_*i*_,*e*_*i*_], i.e., *I*_*ij*_=*∅*, or *I*_*ij*_∈[*s*_*i*_,*e*_*i*_] and |*I*_*ij*_|=1 for some machine *M*_*k*_. Let *a*(*T*_*ij*_)=*k* indicate that task *T*_*ij*_ is assign to machine *M*_*k*_. The empty assignment *I*_*ij*_=*∅* indicates that the task is dropped. If a task is completed, then it has a non-empty assignment on a certain machine such that the assigned sub-interval does not overlap (or conflict) with any assignment on the same machine. We assume *s*_*i*_ and *e*_*i*_ are integers. One unit of time is called a *slot* in this paper. That is, a task needs to take one slot on a machine to be completed. We only consider the problem where each task *T*_*ij*_ has a processing time of length 1. Tasks with arbitrary processing times will be studied in the future work.

We consider a situation where the jobs belong to different users, and the users are always willing to encourage the data centers to complete all their tasks. Therefore, job *J*_*i*_ has an extra utility *σ*_*i*_ in this paper. If all the tasks of job *J*_*i*_ are completed, then the utility gain of this job is the sum of the utility of the tasks included in *J*_*i*_ and the extra utility *σ*_*i*_, i.e. $u(i)={\sum \nolimits }_{j=1}^{n_{i}}u_{ij}+\sigma _{i}$. Otherwise, even one task of a job is not completed, the utility gain is the sum of the utility of the completed tasks, without the extra job utility, i.e. $u(i)={\sum \nolimits }_{j=1,I_{ij}\neq \emptyset }^{n_{i}}u_{ij}$.

### Problem statement

Our problem is to find an assignment of all the tasks of *n* jobs to *m* machines such that the total utility is maximized satisfying that (a) a machine can only process one task at a time, (b) one task can only be processed at one machine, and it cannot be split any more, but the tasks of a job can be assigned to multiple machines. Then, we have

1 $$ \max{\sum\limits_{i=1}^{n}u(i)}  $$

s.t.

2 $$ I_{ij} \subseteq [s_{i},e_{i}]\text{for each task }  $$

3 $$ |I_{ij}| =\{0, 1\}\,\text{for each task }  $$

4 $$ a(T_{ij}) =\{1,2,\dots, m\}\,\text{for each task }  $$

5 $$ I_{ij} \cap I_{i^{\prime} j^{\prime}}=\emptyset\, \text{and }a(T_{ij}) = a(T_{i^{\prime} j^{\prime}})\,\text{for every two tasks }  $$

It is easy to find that this problem is NP-complete. Consider a simple instance where there is only one machine in the problem, the processing interval of each job is [0,*T*], each job *J*_*i*_ consists of *n*_*i*_ one-unit tasks, each job has extra utility *σ*_*i*_, and all the tasks have no utility, i.e. *u*_*ij*_=0, then the problem is to find an assignment within the processing interval while maximizing the utility gain, which is equivalent to the well-known Knapsack problem which is NP-complete. For simplicity, the single-machine problem where there is only one machine can be used is first studied and then followed by the general case where there are multiple machines.

## Algorithm design for single-machine problem

We first consider a simpler instance of this problem where there is only one machine. As stated in the previous section, even the single-machine problem is NP-complete. Therefore, we present a 2-approximation algorithm in this section. The main idea of this algorithm is to solve the problem in two steps. The first step is to solve the single-machine problem without considering extra job utility. The second step is to solve the single-machine problem by only considering the extra job utility. The final result of the single-machine problem can then be obtained by combining the results derived from the two steps.

### Problem without extra job utility

In this step, we do not consider the extra job utility. Therefore, given *n* jobs, each job *J*_*i*_ has a processing interval [*s*_*i*_,*e*_*i*_], each job *J*_*i*_ consists of *n*_*i*_ one-unit-length tasks, and each task *T*_*ij*_ has utility *u*_*ij*_. This step is to find an assignment with the maximum utility gain in a single machine.

We first consider a special case where the utility of each task is 1. Then, the problem is to schedule as many tasks as possible. We introduce the earliest ending time first algorithm which is also called Earliest Deadline First (EDF) in other works and show that the EDF algorithm schedules the maximum number of tasks.

The EDF method always schedules the job with the earliest ending time first. Let *J*_*i*_ be the job with the earliest ending time. EDF scans the processing interval of *J*_*i*_ from *s*_*i*_ to *e*_*i*_ and schedules the tasks of *J*_*i*_ one by one to the unused slots. A slot is *unused* if no tasks are scheduled to this slot. If all the slots in [*s*_*i*_,*e*_*i*_] are scanned, or all the tasks of *J*_*i*_ are scheduled, EDF begins to schedule the next job *J*_*i*+1_.



**Fig. 1:**
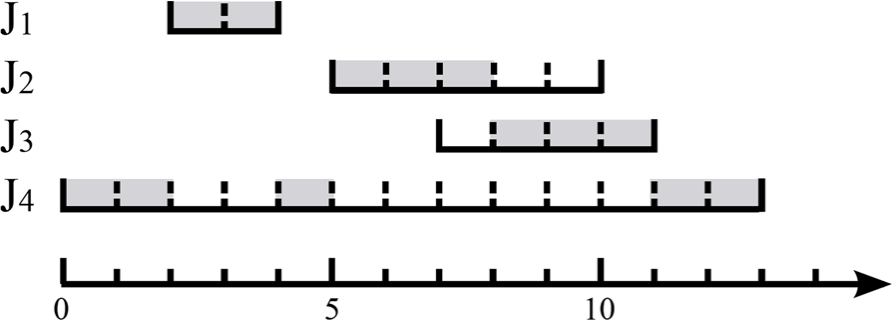
An example for the EDF algorithm

Figure 1 shows an example for the EDF algorithm. In this example, there are four jobs. *J*_1_’s processing interval is [2,4], and it has two tasks. *J*_2_’s processing interval is [5,10], and it has three tasks. *J*_3_’s processing interval is [7,11], and it has three tasks. *J*_4_’s processing interval is [0,13], and it has five tasks. The EDF algorithm schedules the job with the earliest ending time, and the tasks of a job are always scheduled as early as possible in their processing interval. As illustrated in Fig. 1, the gray slots represent the scheduled tasks of the four jobs.

#### **Theorem 1**

The EDF algorithm schedules the maximum number of tasks.

#### *Proof*

We prove the theorem by induction. Let *n*=1, i.e., there is only one job, then it is easy to find that the EDF algorithm schedules the maximum number of tasks.

Assume the EDF algorithm schedules the maximum number of tasks when *n*=*k*.

Now, we prove that the theorem is correct when *n*=*k*+1. If all the tasks of *J*_*k*+1_ can be scheduled in its processing interval, then the theorem is correct. Otherwise, not all the tasks of *J*_*k*+1_ can be scheduled, then there are two cases as follows.

1) If all the scheduled tasks of *J*_1_ to *J*_*k*_ are within the processing interval [*s*_*k*+1_,*e*_*k*+1_], it indicates that *s*_*k*+1_≤min_1≤*i*≤*k*_{*s*_*i*_} and max_1≤*i*≤*k*_{*e*_*i*_}≤*e*_*k*+1_, then all the slots within [*s*_*k*+1_,*e*_*k*+1_] are used.

2) Otherwise, some tasks of *J*_1_ to *J*_*k*_ are scheduled before *s*_*k*+1_, and some tasks of *J*_1_ to *J*_*k*_ are scheduled within [*s*_*k*+1_,*e*_*k*+1_]. No tasks are scheduled after *e*_*k*+1_ because the ending time max_1≤*i*≤*k*_{*e*_*i*_}≤*e*_*k*+1_. We only need to consider whether the scheduled tasks of *J*_1_ to *J*_*k*_ within [*s*_*k*+1_,*e*_*k*+1_] can be moved before *s*_*k*+1_. If we can, then more tasks of *J*_*k*+1_ can be scheduled. However, the EDF algorithm always schedules the tasks as earlier as possible; therefore, it is impossible to move some of these scheduled tasks earlier.

It completes the proof. □

For simplicity of illustration, we explain the meaning of *link* and *reaching* which will be used frequently later. Both link and reach are defined towards the tasks/jobs which are not dropped (the scheduled tasks/jobs). In this paper, the links are directed.

**Fig. 2:**
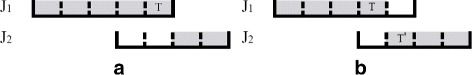
**a**, **b** Job link examples

**Fig. 3:**
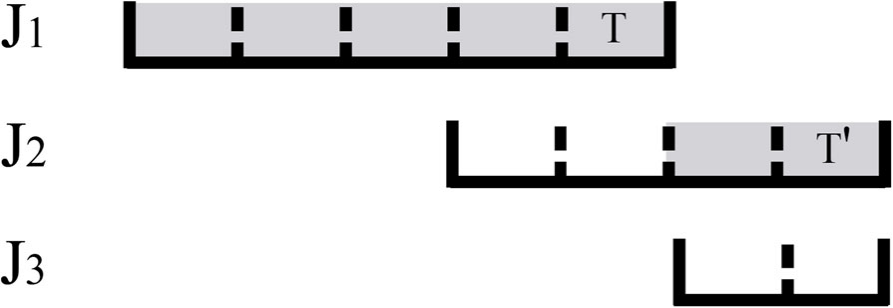
An example for link and reaching

**Fig. 4:**
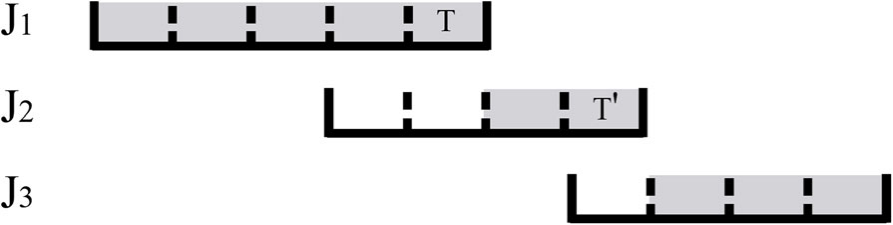
An example for reaching

1) The scheduled tasks of the same job link to each other. We call this the task link.

2) A scheduled job *J*_*i*_ links to another scheduled job *J*_*j*_ if there exists a scheduled task *T* of *J*_*i*_ which is scheduled within the processing interval of *J*_*j*_. We call this the job link and call *T* the relay task. Figure 2 shows some job link examples. In this figure, the gray slots represent the scheduled tasks. In Fig. 2a, *J*_1_ links to *J*_2_ since there is a task *T* belonging to *J*_1_ which is scheduled within the processing interval of *J*_2_. In Fig. 2b, *J*_1_ and *J*_2_ link to each other. Because jobs are scheduled one by one, there may be no task scheduled for the current job. As shown in Fig. 3, no tasks of *J*_3_ are scheduled; however, *J*_2_ still links to *J*_3_ because task *T* ^′^ of *J*_2_ is scheduled within the processing interval of *J*_3_.

3) Scheduled task *T* can reach *T* ^′^ belonging to another job when there exists a job link sequence $\langle J(T),J_{x_{1}},J_{x_{2}}\ldots,J_{x_{k}},J(T')\rangle $ where *J*(*T*) links to $J_{x_{1}}$, $J_{x_{i}}$ links to $J_{x_{i+1}}$, and $J_{x_{k}}$ links to *J*(*T* ^′^). *J*(*T*) denotes the job including *T*. Figure 4 shows that *J*_1_ can reach *J*_3_. In this example, any scheduled task of *J*_1_ can reach any scheduled task of *J*_3_. Reaching is defined for task to task, task to job, and job to job.

We now present a EDF-based algorithm (shown in Algorithm 2). Same as the EDF algorithm, Algorithm 2 always schedules the job with the earliest ending time first. Let *J*_*i*_ be the job with the earliest ending time. EDF scans from *s*_*i*_ to *e*_*i*_ and schedules the tasks of *J*_*i*_ one by one to the unused slots. Note that the tasks of *J*_*i*_ are sorted by utility in non-increasing order. Thus, the task of *J*_*i*_ with the largest utility is scheduled first. Let the current task with the largest utility of *J*_*i*_ be *T*_*ij*_. If there is an unused slot within [*s*_*i*_,*e*_*i*_], *T*_*ij*_ is scheduled to the first unused slot in [*s*_*i*_,*e*_*i*_]. If there is no unused slot within [*s*_*i*_,*e*_*i*_], then find the scheduled task with the least utility and implement a recursive replacement. The replacement is run towards the link sequence from the task with the least utility to the current job, and vice versa. That is, the replacement can also run in a reversed order of the link sequence. In the recursive replacement towards the link sequence, a prior relay task is always replaced with a later relay task. 

**Fig. 5:**
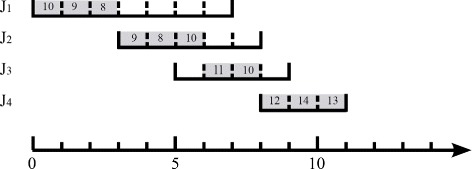
An example for the EDF-based algorithm

Figure 5 shows an example for the EDF-based algorithm. In this example, there are 4 jobs. *J*_1_’s processing interval is [0,7] and it has 5 tasks with utility 10, 9, 8, 7, and 6, respectively. *J*_2_’s processing interval is [3,8], and it has 3 tasks with utility 10, 9, and 8, respectively. *J*_3_’s processing interval is [5,9], and it has 2 tasks with utility 11 and 10, respectively. *J*_4_’s processing interval is [8,11], and it has 3 tasks with utility 14, 13, and 12, respectively. The EDF-based algorithm always schedules the job with the earliest ending time first. In the first iteration, the EDF-based algorithm schedules the 5 tasks of *J*_1_ to slots 1–5, respectively. In the second iteration, the EDF-based algorithm schedules the 3 tasks of *J*_2_ to slots 6–8, respectively.

**Fig. 6:**
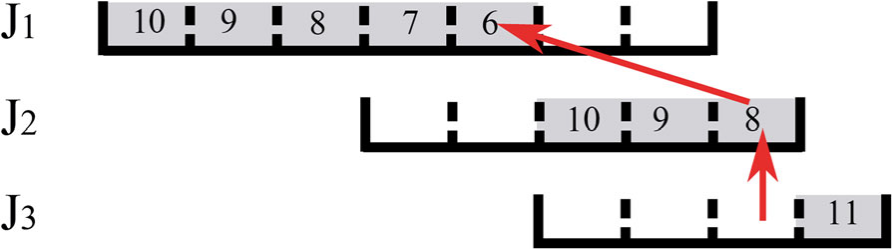
Third interation, for example Fig. 5

In the third iteration, the EDF-based algorithm schedules *J*_3_’s task with a larger utility of 11 to slot 9, and then, it finds that there is no slot for *J*_3_’s task with smaller utility of 10. The algorithm then finds the task with the smallest utility which can reach *J*_3_. The found task is the task of *J*_1_, and its utility is 6. In Fig. 6, the red links show a link sequence for the reachability from the task with utility 6 of *J*_1_ to *J*_3_. The tasks pointed by the red arrows are the relay tasks. Every time a relay task is replaced by a later relay task in the link sequence. The algorithm replaces *J*_1_’s task with utility 6 by *J*_2_’s task with utility 8. In the recursive replacement, *J*_2_’s task with utility 8 is replaced by *J*_3_’s task with utility 10.

**Fig. 7:**
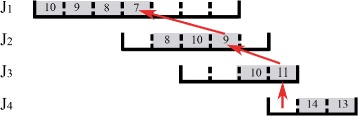
Fourth interation, for example Fig. 5

In the fourth iteration, the EDF-based algorithm schedules *J*_4_’s tasks with utility 14 and 13 respectively, and then, there is no slot for *J*_4_’s task with utility 12. The algorithm then finds the task with the smallest utility which can reach *J*_4_. The found task is the task of *J*_1_, and its utility is 7. In Fig. 7, the red links show a link sequence for the reachability from the task with utility 7 of *J*_1_ to *J*_4_. The tasks pointed by the red arrows are the relay tasks. Every time a relay task is replaced by a later relay task in the link sequence. The algorithm replaces *J*_1_’s task with utility 7 by *J*_2_’s task with utility 9. In the recursive replacement, *J*_2_’s task with utility 9 is replaced by *J*_3_’s task with utility 11, and *J*_3_’s task with utility 11 is replaced by *J*_3_’s task with utility 12.

The final schedule is shown in Fig. 5. The algorithm schedules the maximum number of tasks achieving the maximum total utility. In Fig. 5, the gray slots have been scheduled with tasks, and the numbers on the gray slots represent task utility.

#### **Theorem 2**

The EDF-based algorithm is optimal.

#### *Proof*

Theorem 1 proves that the EDF algorithm schedules the maximum number of tasks. We now only need to prove that the EDF-based algorithm maximizes the total utility of the scheduled tasks. We also prove it by induction.

Let *n*=1, that is, there is only one job. It is easy to find that the EDF-based algorithm maximizes the total utility of the scheduled task because the EDF-based algorithm always schedules the task with the largest utility first.

Assume the EDF-based algorithm maximizes the total utility of the scheduled tasks when there are *k* jobs, i.e., *n*=*k*.

Next, we prove that the theorem is correct when *n*=*k*+1. Then, we need to prove the EDF-based algorithm obtains the optimal result for *J*_*k*+1_.

If all the tasks of *J*_*k*+1_ can be scheduled in its processing interval, then the theorem is correct.

Otherwise, if not all the tasks of *J*_*k*+1_ can be scheduled, then the EDF-based algorithm first schedules as many tasks with the largest utility as possible to the unused slots. As stated in Theorem 1, the EDF algorithm cannot schedule more tasks. Therefore, it should determine whether to schedule the remaining tasks of *J*_*k*+1_ or not. For the remaining tasks, the EDF-based algorithm always uses them to replace the tasks with smaller utility. Without loss of generality, let *T* ^′^ be the task with the largest utility in the remaining tasks and its utility be *u* ^′^. Let *T* be the task with the least utility that has been scheduled, and its utility be *u*. If *u*<*u* ^′^, then the largest utility gain is achieved if *T* is replaced by *T* ^′^. The utility gain is *u* ^′^−*u*_1_+*u*_1_−*u*_2_+*u*_2_−…−*u*_*k*_+*u*_*k*_−*u*=*u* ^′^−*u*, where {*u*_*k*_,*u*_*k*−1_,…,*u*_1_} are the utility of the relay tasks in the link sequence from *T* to *T* ^′^. Because *u* is the minimum, *u* ^′^−*u* is maximum. This property holds for the remaining tasks. It completes the proof. □

The time complexity of the EDF-based algorithm is *O*(*n* ^2^ *r* ^2^), where *n* is the number of jobs and *r* is the maximized number of the tasks of a job. In the EDF-based algorithm, the jobs are scheduled according to the ending time one by one. For the tasks of each job, the algorithm needs to find a task with the smallest utility that has already been scheduled. It needs *O*(*n* *r*) time to search the task in a directed graph constructed by the link relation for tasks to tasks, tasks to jobs, and jobs to jobs. It also needs *O*(*n*) time to update the graph any time the graph is changed, i.e. a replacement is implemented.

6 $${} {{\begin{aligned} & C_{i}(s,\sigma)=\min\\& \left\{\!\!\!\!\! \begin{array}{rl} &C_{i-1}(s,\sigma)\\ &\max\{s_{i},C_{i-1}(s,\sigma-\sigma_{i})\}+n_{i}\\ &\min_{s',\sigma'}\{C_{i-1}(s',\sigma')+\max\{0,n_{i}-s'+s_{i}+P_{i-1}(s,s',\sigma-\sigma_{i}-\sigma')\}\} \end{array} \right. \end{aligned}}}  $$

7 $${}  {{\begin{aligned} &P_{i-1}(s,s',\sigma^{\prime\prime})=\min\\ &\left\{ \begin{array}{rl} &P_{i-1}(s^{+},s',\sigma^{\prime\prime})\\ &\min_{0< \sigma' \le \sigma^{\prime\prime}}\{\max\{0,C_{i-1}(s,\sigma')-s_{i}+P_{i-1}(s^{\prime\prime},s',\sigma^{\prime\prime}-\sigma')\} \end{array} \right. \end{aligned}}}  $$

### Problem with only extra job utility

Now, we address the problem where each job has only extra utility and every task does not have utility. If all the tasks of a job are scheduled, then the job obtains the extra utility. Even one task of a job is not scheduled, the job loses the extra utility. This problem is similar to the one studied in [16] where given *n* jobs with arbitrary processing time, release dates and due dates, and the job can be scheduled preemptively; the objective is to minimize the sum of the weights of the later jobs. The scheduling of our problem is not preemptive, but the processing time of each task is one unit of time. The preemptive scheduling and a task with one unit of processing time in our problem are similar. Therefore, minimizing the sum of the weights of the later jobs is the same as maximizing the sum of the utility of the scheduled jobs in our problem. The authors in [16] give a pseudo polynomial time Dynamic Programming (DP) algorithm. We also adopt this algorithm to solve the problem. The DP formulations are represented by Eqs. (6) and (7).

Given a job set *J*, let $s(J)=\min _{J_{i}\in J}\{s_{i}\}$ be the minimum starting time of *J*, $p(J)={\sum \nolimits }_{J_{i}\in J}n_{i}$ be the total processing time of *J*, $\sigma (J)=\sum _{J_{i}\in J}\sigma _{i}$ be the total extra utility of *J*, and *c*(*J*) be the time the last job in *J* is completed in an EDF schedule.

One can refer to article [16] for the detailed algorithm. As stated in that work, assume the jobs are ordered by the ending time in non-decreasing order. Let *s* be a starting time, and *σ* be an integer representing utility. *C*_*i*_(*s*,*σ*) is defined as the minimum value of *c*(*J*) with respect to feasible set *J*⊆{*J*_1_,*J*_2_,…,*J*_*i*_}, with *s*(*J*)≥*s* and *σ*(*J*)≥*σ*. If there is no such feasible set *J*, then *C*_*i*_(*s*,*σ*)=+*∞*. Accordingly, the final result that maximizing the weight of a feasible set is given by the largest value of *σ* such that *C*_*n*_(*s*_min_,*σ*) is finite, where *s*_min_= min1≤*i*≤*n*{*s*_*i*_}.

If job *J*_*i*_ cannot be contained in a feasible set *J*, i.e. *s*_*i*_<*s*(*J*), then *C*_*i*_(*s*,*σ*)=*C*_*i*−1_(*s*,*σ*).

Otherwise, if job *J*_*i*_ can be contained in the feasible set *J*, there exists two cases.

In the first case, job *J*_*i*_ starts after *c*(*J*−{*J*_*i*_}). Either *c*(*J*−{*J*_*i*_})≤*s*_*i*_, then *C*_*i*_(*s*,*σ*)=*s*_*i*_+*n*_*i*_; or *c*(*J*−{*J*_*i*_})>*s*_*i*_ and the scheduled tasks in the interval [*s*_*i*_,*c*(*J*−{*J*_*i*_})] are continuous for *J*−{*J*_*i*_}, then *C*_*i*_(*s*,*σ*)=*C*_*i*−1_(*s*,*σ*−*σ*_*i*_)+*n*_*i*_. Thus, *C*_*i*_(*s*,*σ*)= max{*s*_*i*_,*C*_*i*−1_(*s*,*σ*−*σ*_*i*_)}+*n*_*i*_.

In the second case, job *J*_*i*_ starts before *c*(*J*−{*J*_*i*_}), which indicates there is an idle time between *s*_*i*_ and *c*(*J*−{*J*_*i*_}). Let *J* ^′^ be the last set of jobs scheduled continuously before *c*(*J*−{*J*_*i*_}) for *J*−{*J*_*i*_}. Then, *c*(*J* ^′^)=*C*_*i*−1_(*s*(*J* ^′^),*σ*(*J* ^′^)). Let it be *c*(*J* ^′^)=*C*_*i*−1_(*s* ^′^,*σ* ^′^) for simplicity.

Let *P*_*i*−1_(*s*,*s* ^′^,*σ* ^″^) be the minimum number of tasks scheduled between *s*_*i*_ and *s* ^′^, with respect to feasible set *J* ^″^⊆{*J*_1_,*J*_2_,…,*J*_*i*−1_} with *s*(*J* ^″^)≥*s*, *c*(*J* ^″^)≤*s* ^′^, and *σ*(*J* ^″^)≥*σ* ^″^. Note that it is the minimum number of tasks scheduled in interval [*s*_*i*_,*s* ^′^], rather than [*s*,*s* ^′^]. Then, the number of slots available for job *J*_*i*_ between *s*_*i*_ and *s* ^′^ can be represented as *s* ^′^−*s*_*i*_−*P*_*i*−1_(*s*,*s* ^′^,*σ*−*σ*_*i*_−*σ* ^′^). Thus, the completing time *C*_*i*_(*s*,*σ*)=*C*_*i*−1_(*s* ^′^,*σ* ^′^)+ max{0,*n*_*i*_−*s* ^′^+*s*_*i*_+*P*_*i*−1_(*s*,*s* ^′^,*σ*−*σ*_*i*_−*σ* ^′^)}.

Enumerate every *s* ^′^ and *σ* ^′^. We can get *C*_*i*_(*s*,*σ*)= min*s* ^′^>*s*,*σ* ^′^<*σ*{*C*_*i*−1_(*s* ^′^,*σ* ^′^)+ max{0,*n*_*i*_−*s* ^′^+*s*_*i*_+*P*_*i*−1_(*s*,*s* ^′^,*σ*−*σ*_*i*_−*σ* ^′^)}}. The enumeration of *s* is among all the starting times of the jobs, rather than among all the possible times, which can drastically reduce the computation complexity.

The computation of *P*_*i*−1_(*s*,*s* ^′^,*σ* ^″^) is as follows. Let *J* ^″^⊆{*J*_1_,*J*_2_,…,*J*_*i*−1_} be the set of jobs which realize *P*_*i*−1_(*s*,*s* ^′^,*σ* ^″^). Then, there exists two cases.

If *s*(*J* ^″^)>*s*, then *P*_*i*−1_(*s*,*s* ^′^,*σ* ^″^)=*P*_*i*−1_(*s*(*J* ^″^),*s* ^′^,*σ* ^″^). Enumerate every *s* ^+^>*s* and find the minimum one, then $P_{i-1}(s,s',\sigma '')=\min _{s^{+}>s}\{P_{i-1}(s^{+},s',\sigma '')\}$.

Otherwise, if *s*(*J* ^″^)=*s* and the scheduling of *J* ^″^ is not continuous, let *J* ^′^ be the first set of jobs which run continuously and *s*(*J* ^′^)=*s*, then the total number of tasks scheduled within [*s*_*i*_,*C*_*i*−1_(*s*,*σ*(*J* ^′^))] is max{0,*C*_*i*−1_(*s*,*σ*(*J* ^′^))−*s*_*i*_}. We now need to compute the number of tasks which can be scheduled within [*C*_*i*−1_(*s*,*σ*(*J* ^′^)),*s* ^′^]. It is easy to find that *P*_*i*−1_(*s*,*s* ^′^,*σ* ^″^) can be represented as max{0,*C*_*i*−1_(*s*,*σ*(*J* ^′^))−*s*_*i*_}+*P*_*i*−1_(*s* ^″^,*s* ^′^,*σ* ^″^−*σ*(*J* ^′^)), where *s* ^″^ is the minimum starting time greater than or equal to *C*_*i*−1_(*s*,*σ*(*J* ^′^)). For simplicity, let *σ* ^′^=*σ*(*J* ^′^). Enumerate every *σ* ^′^. We have *P*_*i*−1_(*s*,*s* ^′^,*σ* ^″^)= min0<*σ* ^′^≤*σ* ^″^{max{0,*C*_*i*−1_(*s*,*σ* ^′^)−*s*_*i*_}+*P*_*i*−1_(*s* ^″^,*s* ^′^,*σ* ^″^−*σ* ^′^)}.

The initial conditions are shown in Eqs. (8) to (11).

8 $$  C_{0}(s,0)=s, \text{~~~~~~~~~~~~~~~~~~for all starting time }s  $$

9 $$  C_{0}(s,w)=+\infty, \text{for all starting time}\, s\, \text{and}\, w>0  $$

10 $$  P_{j-1}=(s,s',0)=0, \text{~~~~~~~~~~~~~for}\, j=1,2,\ldots,n  $$

11 $$  P_{0}(s,s',\sigma^{\prime\prime})=+\infty, \text{~~~~~~~~~~~~~~~~~~~~~~for}\, \sigma^{\prime\prime}>0  $$

The time and space complexities for this DP algorithm are *O*(*n* ^3^ *σ* ^2^) and *O*(*n* ^2^ *σ*), respectively, where *n* is the number of the jobs and *σ* is the sum of the utility of the jobs. It can be easily found that the time complexity of the DP algorithm is pseudo polynomial because the DP formula includes an integer input *σ* which can be extremely large in real systems. Therefore, we provide a theoretical approximation solution for this problem.

### Approximation algorithm for single-machine problem

The main idea of the approximation algorithm is to get two intermediate results using the two algorithms independently and then combine the two results. First, it uses the EDF-based algorithm to solve the problem without extra job utility. Second, it uses the DP algorithm to solve the problem with only extra job utility. Then, it selects a larger one from the two scheduling results. Algorithm APPX1 shows the detailed approximation algorithm. 

#### **Theorem 3**

The APPX1 algorithm is a 2-approximation algorithm.

#### *Proof*

Let OPT be the utility obtained by an optimal solution and ALG be the utility obtained by the APPX1 algorithm. It is easy to find that OPT can be represented as OPT=*u*+*σ*, where *u* is the total utility of the scheduled tasks and *σ* is the total extra utility of the entirely scheduled jobs in an optimal solution. Let *u* ^′^ be the total utility obtained by the EDF-based algorithm, and *σ* ^′^ be the total utility obtained by the DP algorithm. From the earlier analysis, both the EDF-based algorithm for the problem without extra utility and the DP algorithm for the problem with only extra utility are optimal. Thus, *u*≤*u* ^′^ and *σ*≤*σ* ^′^, and then, we have OPT≤*u* ^′^+*σ* ^′^. ALG actually can be represented as ALG≥ max{*u* ^′^,*σ* ^′^}. Therefore, OPT≤2ALG. It completes the proof. □

### An improvement for the DP algorithm

Recall the definition of *C*_*i*_(*s*,*σ*), it is the minimum value of *c*(*J*) with respect to feasible set *J*⊆{*J*_1_,*J*_2_,…,*J*_*i*_}, with *s*(*J*)≥*s* and *σ*(*J*)≥*σ*. It can be found that, in the DP recursion formula *C*_*i*_(*s*,*σ*), the parameter *σ* is build only on the extra utility of the scheduled jobs, but does not consider the utility of the tasks of the scheduled jobs. This can be improved by computing *C*_*i*_(*s*,*u*) where the paremeter is build on the total utility of the scheduled jobs. Let $u_{i}={\sum \nolimits }_{i=1}^{n_{i}}u_{ij}+\sigma _{i}$. Use *u*_*i*_ to replace *σ*_*i*_ in the DP formula, then *C*_*i*_(*s*,*u*) represents the minimum value of *c*(*J*) with respect to feasible set *J*⊆{*J*_1_,*J*_2_,…,*J*_*i*_}, with *s*(*J*)≥*s* and *u*(*J*)≥*u*, where *u*(*J*) includes the utility of all the tasks of the jobs in *J* and the extra utility of the jobs in *J*. Such modification consider the extra utility of the scheduled jobs and also the utility of the tasks of the scheduled jobs. It can improve the result derived by the DP algorithm when the utility of the tasks takes a large proportion comparing with the extra utility. However, when the extra utility takes a large proportion (for example, in a worst case where all the tasks have no utility, the jobs have only the extra utility), the improvement is little. Algorithm APPX1’s use of such modification of the DP algorithm cannot improve the approximation ratio, but may improve the results in many scenarios.

## Algorithm design for multi-machine problem

The solution for the multi-machine problem is similar to the single-machine problem. It also includes two steps. The first step is to schedule the tasks without considering the extra utility of the jobs. The second step is to schedule the jobs only considering the extra utility of the jobs. And finally, select a better schedule from the two steps.

### Problem without extra utility

The EDF-multi-algorithm is similar to the one for the single-machine problem. The difference is that every time the algorithm needs to schedule a task, it finds the earliest unused slot in the task’s processing interval among all the machines, while the algorithm for the single-machine problem just needs to search in one machine. If there is no unused slot, the replacement function also finds the task with the smallest utility that has been scheduled among all the machines. The detailed algorithm is shown in Algorithm 4. The obtained result is optimal for the problem without extra utility. 

### Problem with only extra utility

We design an algorithm for the multi-machine problem with only extra utility by adopting the idea of the DP algorithm for the single-machine problem. Given job set *J*, let $s(J)=\min _{J_{i}\in J}\{s_{i}\}$ be the minimum starting time of *J*, $p(J)={\sum \nolimits }_{J_{i}\in J}n_{i}$ be the total processing time of *J*, and $\sigma (J)={\sum \nolimits }_{J_{i}\in J}\sigma _{i}$ be the total extra utility of *J*.

Define *c*(*J*)=〈*t*,*j*〉 as a 2-tuple where *t* is the time and *j* is the number of used machines at *t* that the last job in *J* is completed in an EDF schedule.

Define $\langle t, j\rangle + p = \left \langle t+\left \lfloor \frac {j+p+1}{m} \right \rfloor, (j+p)\mod m\right \rangle $ which represents scheduling *p* tasks from time *t* and after machine *M*_*j*_ continuously. Because there are *m* machines, *m* tasks can be scheduled in each slot. We focus on how many machines are used in slot *t* rather than which machines are used. Without loss of generality, we assume 〈*t*,*j*〉 represent in slot *t*, machine *M*_1_ to *M*_*j*_ are used. Therefore, the ending time after scheduling *p* tasks from time *t* and after machine *M*_*j*_ continuously is $ t+\left \lfloor \frac {j+p+1}{m} \right \rfloor $, and at the ending time, (*j*+*p*) mod *m* machines are used. In this paper, 〈*t*+1,0〉 equals to 〈*t*,*m*〉 representing in slot *t*, all the *m* machines are used.

Define 〈*t*,*j*〉<〈*t* ^′^,*j* ^′^〉 if *t*<*t* ^′^ or *t*=*t* ^′^ and *j*<*j* ^′^. It represents that 〈*t*,*j*〉 is earlier than 〈*t* ^′^,*j* ^′^〉. 〈*t*,*j*〉=〈*t* ^′^,*j* ^′^〉 only if *t*=*t* ^′^ and *j*=*j* ^′^.

We can regard the scheduling process as putting tasks to a 2-dimensional array from top to bottom and from left to right. Given a starting place 〈*s*,*j*〉, the tasks can be scheduled 〈*s*,*j*+1〉 to 〈*s*,*m*〉, 〈*s*+1,1〉 to 〈*s*+1,*m*〉, 〈*s*+2,1〉 to 〈*s*+2,*m*〉, and so on. Therefore, 〈*t* ^′^,*j* ^′^〉−〈*t*,*j*〉 can be regarded as how many tasks can be put from 〈*t*,*j*〉+1 to 〈*t* ^′^,*j* ^′^〉.

Let *s* be a starting time, *j* be the number of used machines in *s*, and *σ* be an integer representing utility. Similarly to but different from the single-machine problem, *C*_*i*_(〈*s*,*j*〉,*σ*) is defined as the minimum value of *c*(*J*) with respect to feasible set *J*⊆{*J*_1_,*J*_2_,…,*J*_*i*_}, with *s*(*J*)≥*s*, *σ*(*J*)≥*σ* and *j* machines are used in *s*. If there is no such feasible set *J*, then *C*_*i*_(〈*s*,*j*〉,*σ*)=〈+*∞*,+*∞*〉. Accordingly, the final result maximizing the utility of a feasible set is given by the largest value of *σ* such that *C*_*n*_(〈*s*_min_,0〉,*σ*) is finite, where *s*_min_= min1≤*i*≤*n*{*s*_*i*_}.

12 $${}  {{\begin{aligned} &C_{i}(\langle s, 0\rangle,\sigma)=\min\\ &\left\{ \begin{array}{rl} &C_{i-1}(\langle s, 0\rangle,\sigma)\\ &\max\{\langle s_{i}, 0\rangle,C_{i-1}(\langle s, 0\rangle,\sigma-\sigma_{i})\}+n_{i}\\ &\min_{\langle s',0\rangle > \langle s,j\rangle,\sigma'<\sigma}\{C_{i-1}(\langle s',0\rangle,\sigma')+\max\{0,n_{i}-(\langle s', 0\rangle-\langle s_{i},0\rangle)\\& +P_{i-1}(\langle s, 0\rangle,\langle s', 0\rangle,\sigma-\sigma_{i}-\sigma')\}\} \end{array} \right. \end{aligned}}}  $$

13 $${}  {{\begin{aligned} &P_{i-1}(\langle s, 0\rangle,\langle s', 0\rangle,\sigma^{\prime\prime})=\min\\ &\left\{\!\!\!\!\! \begin{array}{rl} &P_{i-1}(\langle s^{+}, 0\rangle,\langle s', 0\rangle,\sigma^{\prime\prime})\\ &\min_{0< \sigma' \le \sigma^{\prime\prime}}\{\max\{0,C_{i-1}(\!\langle s, 0\rangle,\sigma')-\langle s_{i}, 0\rangle\!\}+P_{i-1}(\langle s^{\prime\prime},0\rangle,\langle s', 0\rangle,\sigma^{\prime\prime}-\sigma')\} \end{array} \right. \end{aligned}}}  $$

The dynamic programming recursion formula is shown in Eqs. (12) and (13). We now introduce the recursion formula in details. The definition of *C*_*i*_(〈*s*,0〉,*σ*) whose value is a 2-tuple represent the smallest ending place 〈*t*,*x*〉 where a feasible set *J*⊆{*J*_1_,*J*_2_,…,*J*_*i*_} can complete, while satisfying *u*(*J*)≥*σ*, *s*(*J*)≥*s* and *j* machines are used in *s*.

If *J*_*i*_∉*J*, i.e., *J*_*i*_ cannot be contained in a feasible set *J* satisfying the constraint, *C*_*i*_(〈*s*,0〉,*σ*)=*C*_*i*−1_(〈*s*,0〉,*σ*).

Let us consider the situation *J*_*i*_∈*J*, where *J*_*i*_ is contained in a feasible set *J* satisfying the constraint. There are two cases as follows.

Case 1: Job *J*_*i*_ is apparent to start after 〈*s*_*i*_,0〉. If *c*(*J*−{*J*_*i*_})≤〈*s*_*i*_,0〉, or else *c*(*J*−{*J*_*i*_})>〈*s*_*i*_,0〉 and *J*−{*J*_*i*_} are scheduled continuously from 〈*s*_*i*_,0〉 to *c*(*J*−{*J*_*i*_}), then *C*_*i*_(〈*s*,0〉,*σ*)= max{〈*s*_*i*_,0〉,*C*_*i*−1_(〈*s*,0〉,*σ*−*σ*_*i*_)}+*n*_*i*_.

Case 2: Job *J*_*i*_ is apparent to start after 〈*s*_*i*_,0〉. But the tasks scheduled between 〈*s*_*i*_,0〉 and *c*(*J*−{*J*_*i*_}) are not continuous. That is, some tasks of job *J*_*i*_ can be scatterred between 〈*s*_*i*_,0〉 and *c*(*J*−{*J*_*i*_}) rather than after *c*(*J*−{*J*_*i*_}). As stated in [16], the EDF method schedules the tasks as the form of periods of continuous processing. A period of continuous processing is called a block. We consider the scheduling of *J*−{*J*_*i*_} in the DP algorithm. Let the starting time of the last block in *J*−{*J*_*i*_} be 〈*s* ^′^,0〉, and the utility of the last block in *J*−{*J*_*i*_} be *σ* ^′^, then the ending time of the last block is *C*_*i*−1_(〈*s* ^′^,0〉,*σ* ^′^)}. Let *P*_*i*−1_(〈*s*,0〉,〈*s* ^′^,0〉,*σ* ^″^) be the minimum amount of processing done between 〈*s*_*i*_,0〉 and 〈*s* ^′^,0〉, with respect to feasible set *J* ^″^⊆{*J*_1_,*J*_2_,…,*J*_*i*−1_} with *s*(*J* ^″^)≥*s*, *c*(*J* ^″^)≤*s* ^′^, and *σ*(*J* ^″^)≥*σ* ^″^, then the number of slots available for job *J*_*i*_ between 〈*s*_*i*_,0〉 and 〈*s* ^′^,0〉 is

〈*s* ^′^,0〉−〈*s*_*i*_,0〉−*P*_*i*−1_(〈*s*,0〉,〈*s* ^′^,0〉,*σ*−*σ*_*i*_−*σ* ^′^).

Then, the completing time *C*_*i*_(〈*s*,0〉,*σ*) can be represented as

*C*_*i*−1_(〈*s* ^′^,0〉,*σ* ^′^)}+ max{0,*n*_*i*_−〈*s* ^′^,0〉+〈*s*_*i*_,0〉+*P*_*i*−1_(〈*s*,0〉,〈*s* ^′^,0〉,*σ*−*σ*_*i*_−*σ* ^′^)}

Enumerating every *s* ^′^ and *σ* ^′^, we can get

*C*_*i*_(〈*s*,0〉,*σ*)= min〈*s* ^′^,0〉>〈*s*,0〉,*σ* ^′^<*σ*{*C*_*i*−1_(〈*s* ^′^,0〉,*σ* ^′^)}+ max{0,*n*_*i*_−〈*s* ^′^,0〉+〈*s*_*i*_,0〉+*P*_*i*−1_(〈*s*,0〉,〈*s* ^′^,0〉,*σ*−*σ*_*i*_−*σ* ^′^)}}.

We now introduce how to realize the the computation of *P*_*i*−1_(〈*s*,0〉,〈*s* ^′^,0〉,*σ* ^″^). Recall the definition of *P*_*i*−1_(〈*s*,0〉,〈*s* ^′^,0〉,*σ* ^″^). It is the minimum number of tasks scheduled between 〈*s*_*i*_,0〉 and 〈*s* ^′^,0〉 satisfying the utility constraint. Assume *P*_*i*−1_(〈*s*,0〉,〈*s* ^′^,0〉,*σ* ^″^) is achieved by a non-empty set *J* ^″^⊆{*J*_1_,*J*_2_,…,*J*_*i*−1_}.

If 〈*s*(*J* ^″^),0〉>〈*s*,*j*〉, then *P*_*i*−1_(〈*s*,0〉,〈*s* ^′^,0〉,*σ* ^″^)=*P*_*i*−1_(〈*s*(*J* ^″^),0〉,〈*s* ^′^,0〉,*σ* ^″^). Therefore, we can enumerate every *s* ^+^>*s* and find the result. The result is the minimum one among $\min _{s^{+}>s}\{P_{i-1}(\langle s^{+},0\rangle,\langle s', 0\rangle,\sigma '')\}$.

Otherwise, 〈*s*(*J* ^″^),0〉=〈*s*,0〉. Let the first block in the solution be *J* ^′^ and the total extra utility of the first block be *σ* ^′^, then the ending time of the first block is *C*_*i*−1_(〈*s*,0〉,*σ* ^′^). Therefore, *P*_*i*−1_(〈*s*,0〉,〈*s* ^′^,0〉,*σ* ^″^) can be represented as max{0,*C*_*i*−1_(〈*s*,0〉,*σ* ^′^)−〈*s*_*i*_,0〉}+*P*_*i*−1_(〈*s* ^″^,0〉,〈*s* ^′^,0〉,*σ* ^″^−*σ* ^′^), where *s* ^″^ is the smallest starting time 〈*s* ^″^,0〉≥*C*_*i*−1_(〈*s*,0〉,*σ* ^′^). Enumerating every *σ* ^′^, we can obtain *P*_*i*−1_(〈*s*,0〉,〈*s* ^′^,0〉,*σ* ^″^)= min0<*σ* ^′^≤*σ* ^″^{max{0,*C*_*i*−1_(〈*s*,0〉,*σ* ^′^)−〈*s*_*i*_,0〉}+*P*_*i*−1_(〈*s* ^″^,0〉,〈*s* ^′^,0〉,*σ* ^″^−*σ* ^′^)}.

The initial conditions are shown in Eqs. (14) to (17).

14 $${}  C_{0}(\langle s, 0\rangle,0)=\left\langle\left\lceil\frac{s+1}{m}\right\rceil, s\, \text{mod}\, m\right\rangle, \text{for all starting time }s  $$

15 $${}  C_{0}(\langle s,0\rangle,w)=+\infty, \text{for all starting time}\, s\, \text{and}\, w>0  $$

16 $$  P_{j-1}=(\langle s,0\rangle,\langle s',0\rangle,0)=0, \text{~~~for}\, j=1,2,\ldots,n  $$

17 $$  P_{0}(\langle s,0\rangle,\langle s',0\rangle,\sigma^{\prime\prime})=+\infty, \text{~~~~~~~~~~~for}\, \sigma^{\prime\prime}>0  $$

An improvement of the DP formula for the single-machine problem can also be used in the multi-machine problem. It cannot improve the result in a worst case where the tasks have no utility, but it can improve the result for many inputs.

### Approximation algorithm for multi-machine problem

The same as single-machine problem, the main idea of the approximation algorithm for the multi-machine problem is to get two intermediate results using the EDF-multi-algorithms and the DP algorithm for the multi-machine problem independently and then combine the two results of the two algorithms. First, it uses the EDF-multi-algorithm to solve the problem without extra job utility. Second, it uses the DP algorithm to solve the problem with only extra job utility. Finally, it selects a larger one from the two scheduling results. Algorithm APPXm shows the detailed approximation algorithm. 

#### **Theorem 4**

The APPXm algorithm is a 2-approximation algorithm.

#### *Proof*

Let OPT be the utility obtained by an optimal solution and ALG be the utility obtained by the APPXm algorithm. It is easy to find that OPT can be represented as OPT=*u*+*σ*, where *u* is the total utility of the scheduled tasks and *σ* is the total extra utility of the entirely scheduled jobs in an optimal solution. Let *u* ^′^ be the total utility derived by the EDF-multi-algorithm and *σ* ^′^ be the total utility obtained by the dynamic programming algorithm. From the earlier analysis, both the EDF-multi-algorithm for the problem without extra utility and the dynamic programming algorithm for the problem with only an extra utility are optimal. Thus, *u*≤*u* ^′^ and *σ*≤*σ* ^′^, then we have OPT≤*u* ^′^+*σ* ^′^. ALG actually can be represented as ALG≥ max{*u* ^′^,*σ* ^′^}. Therefore, OPT≤2ALG. It completes the proof. □

## Simulation result

The simulation result is shown in this section. As the computation complexity of our algorithm is high, especially the DP algorithm; thus, the input of our simulation is set to not too large. The number of machines used in our simulation is at most 5, the number of applications is at most 100, and the number of tasks for each application is at most 5. The utility of each task is a random value. The starting time and the ending time and the extra utility of each application is also randomly generated.

As the optimal result is hard to compute; thus, we use an upper bound result to represent the optimal result. The upper bound is computed by dividing the extra utility of each application into its tasks in proportion, that is task with high utility will be assigned with a high extra utility.

**Fig. 8:**
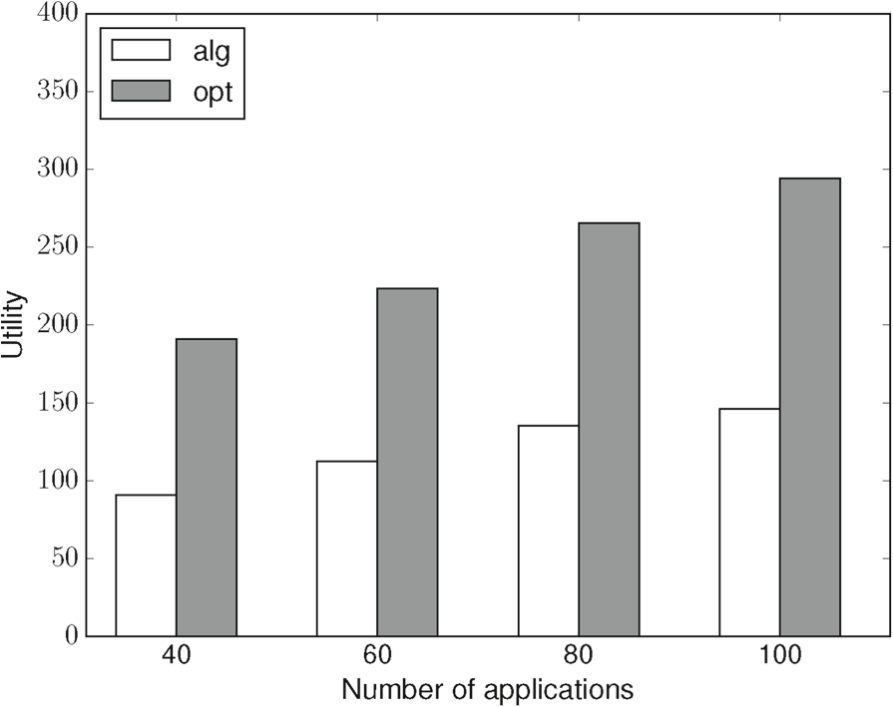
Scheduling with single machine

**Fig. 9:**
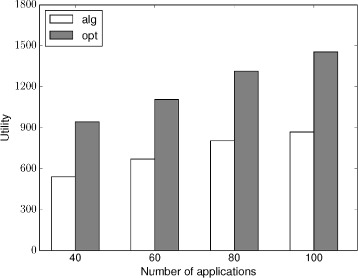
Scheduling with multi-machine

The scheuding result in single machine is shown in Fig. 8. As shown in Fig. 8, the utility that the approximation algorithm get increases as the number of applications increases. However, when the number of application increases to a certain degree, the utility that the approximation algorithm can get increases slower; this is because the single machine is approaching to its maximum computing load. Figure 9 illustrates the scheduling result in five machines. Similar as the single-machine case, the utility that the approximation algorithm can get increase as the number of applications increases. And the increasing rate gets slower as the multiple machines are getting to their maximum load. Because the upper bound we used to represent the optimal result is higher than the real optimal result, therefore, the utility that the approximation algorithm gets can get closer to the optimal result and the difference is much less than two times, which confirms the approximation ratio; thus, the performance is acceptable in our simulation.

## Conclusions

This paper proposes a class of algorithms to solve the problem of maximizing utility for job scheduling where each job consists of multiple tasks. Different from the existing works which either consider job utility or task utility individually, this paper considers both job utility and task utility simultaneously by introducing extra utility for every job. We analyze the complexity of the problem and discuss two sub-problems of scheduling jobs in a single machine and scheduling jobs in multiple machines. We design two 2-approximation algorithms for the sub-problems, and the approximation proofs are also presented. Although the time complexity is pseudo polynomial, we provide a theoretical insight into this problem.
